# Assaying Environmental Nickel Toxicity Using Model Nematodes

**DOI:** 10.1371/journal.pone.0077079

**Published:** 2013-10-07

**Authors:** David Rudel, Chandler D. Douglas, Ian M. Huffnagle, John M. Besser, Christopher G. Ingersoll

**Affiliations:** 1 Department of Biology, East Carolina University, Greenville, North Carolina, United States of America; 2 Columbia Environmental Research Center, U.S. Geological Survey, Columbia, Missouri, United States of America; CSIR-Central Drug Research Institute, India

## Abstract

Although nickel exposure results in allergic reactions, respiratory conditions, and cancer in humans and rodents, the ramifications of excess nickel in the environment for animal and human health remain largely undescribed. Nickel and other cationic metals travel through waterways and bind to soils and sediments. To evaluate the potential toxic effects of nickel at environmental contaminant levels (8.9-7,600 µg Ni/g dry weight of sediment and 50-800 µg NiCl_2_/L of water), we conducted assays using two cosmopolitan nematodes, *Caenorhabditis elegans* and *Pristionchus pacificus*. We assayed the effects of both sediment-bound and aqueous nickel upon animal growth, developmental survival, lifespan, and fecundity. Uncontaminated sediments were collected from sites in the Midwestern United States and spiked with a range of nickel concentrations. We found that nickel-spiked sediment substantially impairs both survival from larval to adult stages and adult longevity in a concentration-dependent manner. Further, while aqueous nickel showed no adverse effects on either survivorship or longevity, we observed a significant decrease in fecundity, indicating that aqueous nickel could have a negative impact on nematode physiology. Intriguingly, *C. elegans* and *P. pacificus* exhibit similar, but not identical, responses to nickel exposure. Moreover, *P. pacificus* could be tested successfully in sediments inhospitable to *C. elegans*. Our results add to a growing body of literature documenting the impact of nickel on animal physiology, and suggest that environmental toxicological studies could gain an advantage by widening their repertoire of nematode species.

## Introduction

Nickel occurs naturally in soils, sediments, and waters and is an essential metal for many organisms, particularly plants and microbes. Substantial levels of nickel are introduced into the environment via volcanic and anthropogenic activities. When released into the environment from human manufacturing waste, nickel, as Ni(II), can assume both soluble and insoluble forms. In aquatic environments, particulate nickel remains close to the source of contamination, but soluble nickel is mobile and can be incorporated into soils and sediments at greater distances. Nickel toxicity in waters and sediments is assumed to be closely related to concentrations of dissolved metal ions. In sediment pore water, concentrations of dissolved metal ions are controlled primarily by nickel binding to sulfides, measured as acid-volatile sulfide or AVS, and organic matter, measured as total organic carbon or TOC [1].

Nickel is harmful to animals when present in excess [2,3]. In humans, it can induce allergic reactions and skin rashes [4–6] and promote respiratory illnesses and cancer [3,7–9]. Many forms of nickel also have been demonstrated to induce carcinoma formation in rodents [2,3,10]. Studies suggest that insoluble forms can be more detrimental to animal life than soluble forms [11,12]. Nevertheless, the toxicity of soluble and sediment-bound nickel and potential toxic effects of nickel at realistic contaminant levels remain unresolved.

Nematodes are ideal for aquatic toxicology studies. They are the most abundant multicellular animals, inhabit nearly every environment, and have a wide array of feeding and life strategies, making them integral parts of most ecosystems. Free-living nematodes occur in high densities in almost every type of soil, sediment, and water, often exceeding a million individuals per cubic meter. Importantly, many are found in soft-sediment benthic environments [13] that are particularly susceptible to anthropogenic contamination. The desire to use nematodes as bio-indicators to assay contaminants in environmental contexts has resulted in much effort by researchers and environmental monitoring agencies to generate standardized assays for field collected sediment, soil, and water samples [14–17]. This is particularly important as the physiological and genetic responses of animals to toxins are subject to complex environmental influences. Environmental differences can either enhance or mitigate a toxins effect, for example, through binding or sequestration. Additionally, phenotypic plasticity or the interactions between the environment and the genome can alter an animal’s physiological response [18–20].

Two nematodes in particular, *Caenorhabditis elegans* and *Pristionchus pacificus*, are advantageous models for environmental toxicology studies. Ecologically, they represent two of the most successful and abundant nematode families (Rhabditidae and Diplogastridae, respectively) [21,22]. Both have preferred habitats that intersect those of humans; *C. elegans* is found in environments rich in rotting organic matter, like human compost heaps and orchards [23], whereas *P. pacificus* occupies a variety of environments in association with beetles [24,25]. In addition to their ecological advantages, these species are well-characterized models for animal development and basic cellular and biochemical processes, with a track record of findings that translate to human health [26]. *Caenorhabditis elegans* and *P. pacificus* are hermaphroditic, and have invariable life cycles when grown at constant temperature. Complete development from laid egg to fertile adult is approximately 78 hours for *C. elegans* and 82 hours for *P. pacificus* at 20°C [27]. During development, both species go through four larval stages (L1-L4 in *C. elegans*; J1-J4 in *P. pacificus*), punctuated by molts of the cuticle to a final adult form. Easily observed organs and tissues, including the vulva, somatic gonad, and germ line, as well as overall size, can be used to characterize development through these stages morphologically. Thus, these two nematodes offer the opportunity to conduct mating controlled tests that span the whole of development in timely four- or five-day assays.

Nematodes have been used for both environmental assays and laboratory based testing of many metals (e.g., cadmium, cobalt, copper, lead, magnesium, manganese, nickel, and zinc) known to be potentially toxic to animals [28–33]. With a few exceptions [34], almost all studies have used either established environmental assays or other laboratory testing methods, and have relied solely upon the model nematode *C. elegans*, ignoring the potential benefits offered by nematode species diversity. Laboratory tests have implicated nickel in affecting adult *C. elegans* body size, generation time, brood size, germ cell viability and a number of other physiological parameters [35–38]. Although sediment assays have been developed, most previous laboratory nickel studies on nematodes and other developmental genetic animal models used high nickel concentrations delivered in simplified media [2,3,35]. Thus many testing agencies remain ambivalent about the effects of lower levels of nickel exposure to animal and human health [2,3,39]. Tests involving lower nickel concentrations characteristic of environmental contamination have yet to be conducted, particularly with respect to developmental effects in the context of endogenous sediments and waters.

Here, we use *C. elegans* and *P. pacificus* to test the effects of nickel in moderately hard test waters and freshwater sediments collected from Midwestern United States watersheds [40]. Our findings contribute to two aspects of the ongoing effort to assess environmental nickel toxicity. First, we analyze the effects on development, health and reproduction throughout the animal’s life cycle. We show that typical environmental contaminant levels of nickel negatively impact the survival of nematodes to adulthood, adult longevity, and fecundity. Second, our results demonstrate the utility of a multi-species approach when using nematodes for environmental assays.

## Materials and Methods

### Nematode Strains, Handling, Synchronization, and Staging

**Figure 1:**
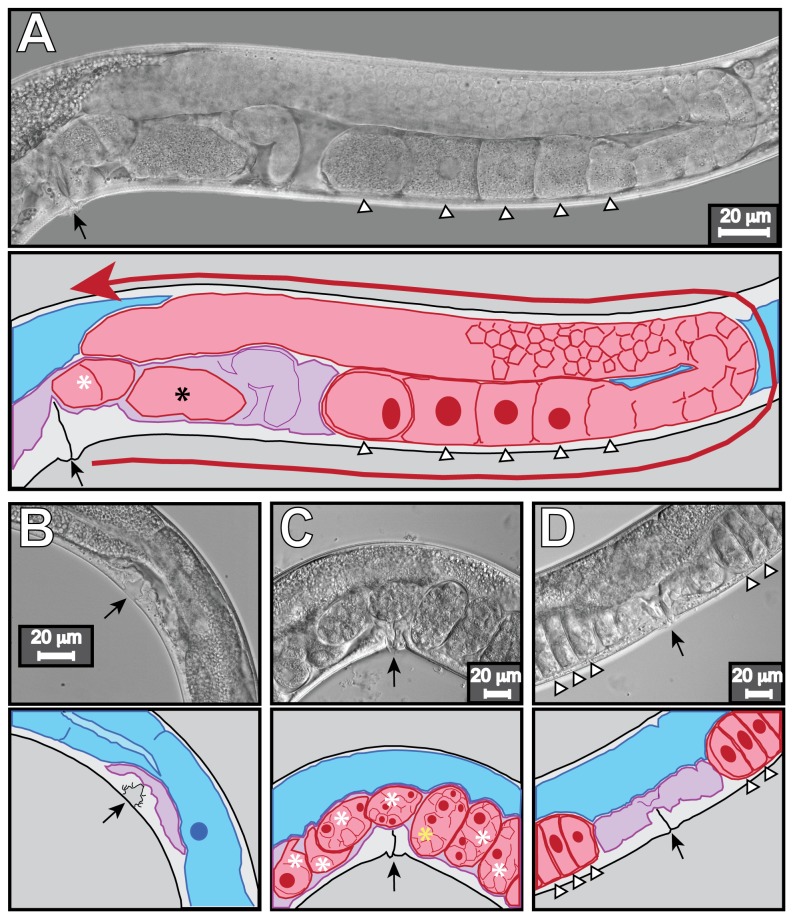
Morphological markers of life-stage and fertility in *C. elegans*. Detailed descriptions of *P. pacificus* vulva and gonad development are available [38,39,42]. Panels include a DIC micrograph on top and a cartoon of the micrograph below. For body orientation, anterior is to the left and dorsal to the top. Black arrows mark the position of the vulva, the egg- laying organ. Asterisks in the cartoons denote developing embryos *in* *utero* and are not shown in the micrograph for clarity. White triangles denote the position of oocytes. Tissues are denoted by color: gut, blue; uterus and spermatheca, purple; germ line (including the germ cells, oocytes, and developing embryos), red. Ovals and circles depict easily seen nuclei within tissues. (A) Young adult hermaphrodite. The entire posterior gonad arm is shown. The red arrow outlines the path of gonad arm extension starting proximal to the vulva and terminating with the arrowhead at the distal tip. As an adult, this animal has made sperm, oocytes, and contains embryos, has two fully reflexed and inflated gonadal arms, and a fully everted vulva with a slit like morphology. The black asterisk denotes an egg in the spermatheca that has just been fertilized but has not developed an eggshell yet. In contrast, L4 larvae never contain embryos as they only begin to produce gametes late in the L4-stage. The L4 gonad arms are smaller and not as inflated, and early in L4 have only reached the dorsal side of the animal, but do not reach the center above the vulva until late in L4. (B) Wild type early L4-larva hermaphrodite detail of uterus and vulva. The vulva has a characteristic “Christmas tree” like shape, it is not everted. The uterus is empty and un-inflated. Gametes have not been produced, the gonad arms are skinny and contain relatively few germ cells making the gonad arms difficult to capture in the same focal plane as the vulva. (C) Wild type adult hermaphrodite detail of uterus and vulva. The uterus is full of multicellular embryos. The gold asterisk denotes an embryo with a clearly visible eggshell. The eggshell is present as an oval around the ball of cells. The embryo and shell are separated by a slim cleared liquid-filled space. (D) Adult fog-*2* female detail of uterus and vulva. *fog-2* females do not produce sperm and contain no embryos. Hence the uterus and spermatheca remain unexpanded. Unfertilized oocytes stack up in the gonad arms and become compressed, giving a “piano key” phenotype. Eventually pressure may push an oocyte into the uterus and the oocyte will be laid, but without an eggshell. Laid oocytes have a “mushy” appearance and remain single celled until decomposition. The edge of a laid embryo has a refractory appearance due to the eggshell.

*Caenorhabditis elegans* laboratory strain N2, *C. elegans* strain JK574: *Cel-fog-2* (*q71*) *LGV*, and *Pristionchus pacificus* laboratory strain PS312 were used. *Cel-fog-2* is gonochoristic and must be maintained through matings. Animals were maintained at 20°C on either Nematode Growth Media (NGM) or K media plates seeded with *Escherichia coli* strain OP50 using standard culture techniques [41]. Animals were age/development synchronized for all tests. Mixed staged cultures were grown on OP 50 bacterial seeded 100mm K media plates. Fully-grown cultures were washed off plates using M9 Buffer, placed into 15 mL conical tubes, and centrifuged at 800 g for 10 minutes at 4°C. The supernatant was drawn off the pellet and 10 mL of fresh M9 added to the conical tube. The nematode/egg pellet was suspended, centrifuged, and washed with M9 twice more for a total of three washes. The cleansed pellet was suspended in 10 mL of a basic hypochlorite solution and agitated for approximately five minutes. The surviving eggs were centrifuged at 1200 rpm after most adult carcasses were dissolved, and the pellet washed three times with fresh M9 buffer. Washed eggs were removed from the conical tube and placed in batches on unseeded K media plates to hatch for 24 hours. Without food, *C. elegans* eggs hatch and developmentally arrest as L1 larvae, and *P. pacificus* as J2 larvae. Nematodes were developmentally staged via observation of the vulva [42,43], somatic gonad [44–46], and germ line [47,48], as described (see Figure 1).

### Sediments and Test Water

**Figure 2:**
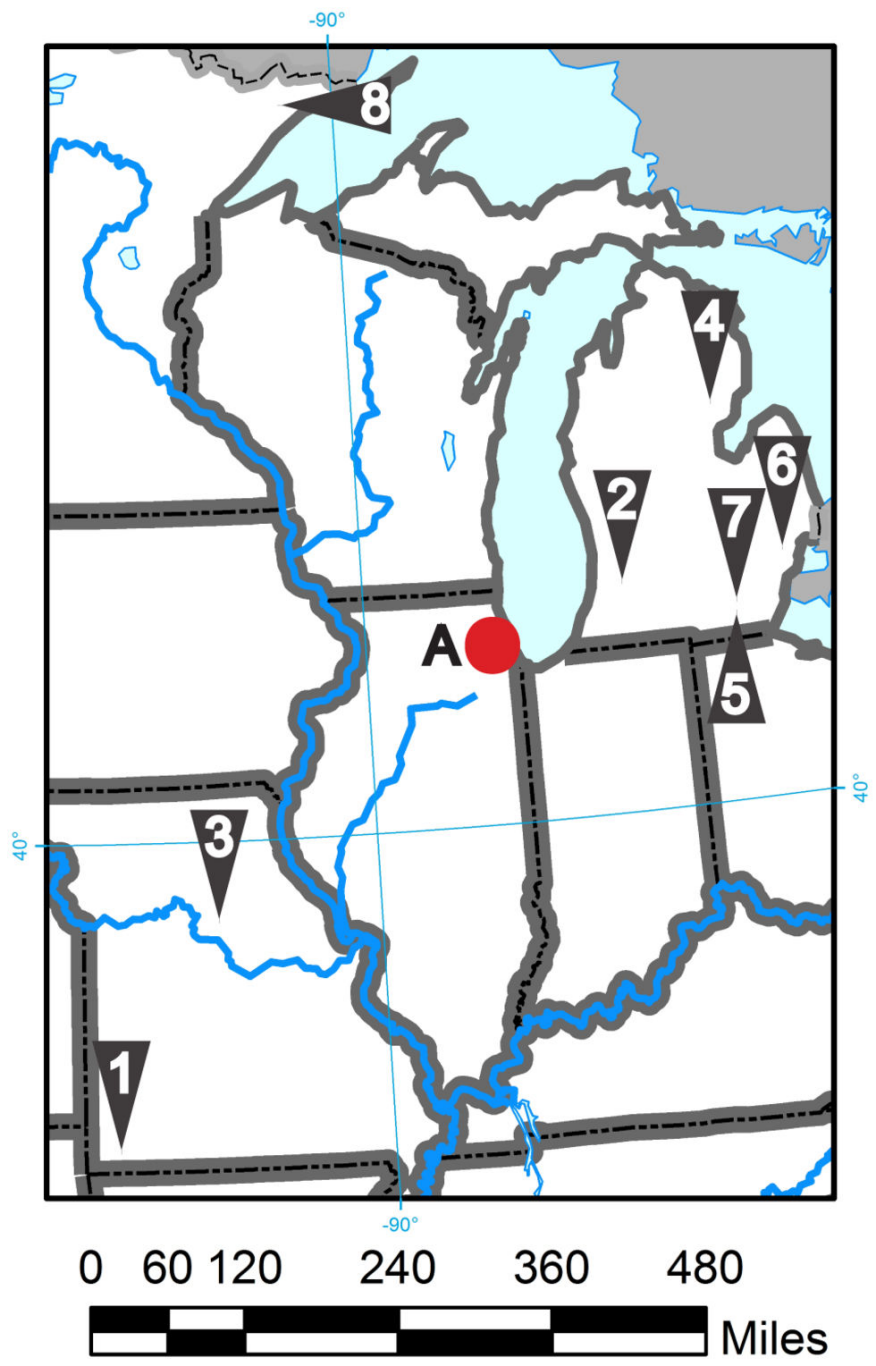
Collection sites for test sediment in the Midwest USA. (1) SR – Spring River, Jasper County, Missouri(2). STJ – St. Joseph River, Michigan(3). P30 – USGS Pond 30 Missouri(4). DOW – Dow Creek Michigan(5). RR2 – Raisin River Site 2, Michigan(6). STM – South Tributary of Mill Creek, Michigan(7). RR3-0 Raisin River Site 3, Michigan(8). WB – West Bearskin Lake Minnesota. Sites are given in order presented in Tables 1-3 and based upon *C. elegans* survivorship performance. The letter A designates the position of Chicago, IL, USA.

Sediments were collected from eight sites in Michigan, Missouri, and Minnesota, USA (Figure 2). Site WB is within a publicly accessible portion of a national forest. Sites RR2 and RR3 are within a publicly accessible state forest. SR, DOW, STJ, and STM sites are from public right-of-way access to the waterway. Site P30 is a pond at the USGS facility in Columbia, MO USA. No specific permits were required for the study sites, but permission for access to the P30 site was granted by Rip Shively, Director, U.S. Geological Survey, Columbia Environmental Research Center. Samples were characterized, and spiked with nickel as reported [40] (Tables 1-4). Sediments contained background levels of many elements, particularly metals such as aluminum, iron, magnesium, manganese, and zinc in levels typically acknowledged as non-harmful (Table 3). Sediments were stored at 4°C in sealed glass jars. Test water was used for both the sediments and water tests. Test water was produced by dilution of well water from the USGS/CERC with de-ionized water to a hardness of 100 mg/L (as CaCO_3_) as reported [40]. Prior to use, test water was titrated to a pH of 7.5 using minimal amounts of HCl and NaOH. For nickel-spiked sediments, treatments were identified by a two-letter sediment ID plus a numeric suffix indicating the un-spiked controls (0) and the five nickel-spike levels (1-5). Nickel spikes increased by a factor of 2 between levels, and the highest nominal concentrations for the SR and WB sediments were 705 and 8,500 µg Ni/g dry weight, respectively [40]. For water-only toxicity tests, nominal NiCl_2_ solutions were 50, 100, 200, 400, and 800µg/L nickel, a range of concentrations chosen to encompass the nickel concentrations likely to occur in the pore water of the nickel-spiked sediments.

**Table 1:** Physico-chemical characteristics of un-spiked sediments.

**Sediment**	pH	ORP (mv)	**TOC (%)**	AVS (µmol/g)	Particle Size (%)			**CEC (meq/100g)**	log Kd	**TR-Ni (µg/g)**
					Clay	Silt	Sand			
**SR**	7.03	-169	**0.40**	0.94	6.9	13.6	79.5	**5.5**	3.56	**8.9**
**STJ**	7.28	-186	**1.9**	3.78	7.9	10.3	81.8	**11.3**	3.979	**8**
**P30**	6.87	-168	**1.8**	12.37	24.2	66.0	9.8	**19.0**	4.248	**14**
**DOW**	6.90	-155	**1.2**	1.04	6.0	7.0	87.0	**6.4**	3.794	**6**
**RR2**	6.98	-188	**3.5**	6.06	8.1	19.8	72.1	**14.5**	4.164	**12**
**STM**	7.14	-189	**8.1**	24.70	8.4	37.5	54.1	**29.1**	4.349	**18**
**RR3**	7.02	-179	**7.2**	7.98	5.9	19.5	74.6	**29.3**	3.857	**9**
**WB**	6.63	-87	**10.40**	38	24.7	68.3	7.0	**44.1**	4.56	**59.7**

Nickel distribution coefficient, Kd = TR-Ni/pore-water Ni

Table modified from Besser et al. 2011.

ORP=oxidation-reduction potential, TOC=total organic carbon, AVS=acid-volatile sulfide, CEC=cation exchange capacity, TR-Ni=total-recoverable nickel

**Table 2:** Major constituents of pore waters from un-spiked sediments.

**Treatment**	**DOC**	**Cl^-^**	**F^-^**	**_-_NO_3_**	**_2-_SO_4_**	**Ca**	**Fe**	**K**	**Mg**	**Mn**	**Na**
**SR**	7	16.7	0.4	<0.08	<0.08	118	9.0	5.6	8.7	10.0	10
**STJ**	32	40	0.9	7.7	18.2	237	10	4.9	67	10	22
**P30**	21	20	<0.08	6.6	<0.08	182	46	7.0	52	6	22
**DOW**	51	79	<0.08	<0.08	<0.08	271	35	7.2	61	8	26
**RR2**	21	19	0.9	7.2	<0.08	189	21	4.4	47	5	10
**STM**	47	85	<0.08	<0.08	<0.08	348	35	10.0	70	4	26
**RR3**	32	27	<0.08	5	<0.08	192	29	4.3	43	7	16
**WB**	30	18.8	0.4	<0.08	80	26	18.0	2.0	6.9	2.9	6.6

All values in mg/L.

Table modified from Besser et al. 2011.

**Table 3:** Total-recoverable element concentrations in un-spiked sediments.

**Sample**	**Al**	**Ba**	**Ca**	**Cr**	**Fe**	**K**	**Mg**	**Mn**	**Na**	**Sr**	**V**	**Zn**
**SR**	12,556	-	1,481	-	7,753	1,598	488	215	-	-	-	54.9
**STJ**	7,212	72	6,731	14	22,260	962	1,875	529	<1000	16	19	39
**P30**	28,947	158	34,450	33	16,268	2,392	3,206	292	<1000	86	48	46
**DOW**	6,965	39	3,682	10	6,468	995	1,692	119	995	17	16	40
**RR2**	7,000	70	56,500	15	10,700	1,500	7,000	425	<1000	50	20	45
**STM**	10,784	172	85,784	15	24,755	2,745	12,892	637	<1000	162	28	64
**RR3**	5,238	95	54,762	10	14,429	476	7,000	905	<1000	110	14	43
**WB**	24,707	-	4,546	-	51,317	5,957	5,368	678	-	-	-	141.1

[All values µg/g dry weight. All samples were below detection limits for Be (<0.5), B (<200), Cd(<2), Co (<5), Cu (<10), Mo (<50), Pb (<50).]

Table modified from Besser et al. 2011.

**Table 4:** Physico-chemical characteristics of nickel-spiked sediments.

**TM**	pH	ORP (mV)	**TOC (%)**	AVS (µmol/g)	Particle Size (%)			**CEC (meq/100 g)**	log Kd	**OW-Ni** (µg/L)	**TR - Ni (mg/kg)**
					Clay	Silt	Sand				
**WB-0**	6.63	-87	**10.40**	38	24.7	68.3	7.0	**44.1**	4.56	**0.93**	**59.7**
**WB-1**	6.62	-79	**11.20**	25	21.1	66.1	12.8	**42.5**	4.37	**4.62**	**156**
**WB-2**	6.65	-85	**9.70**	26	20.6	66.5	12.9	**37.4**	4.24	**8.57**	**369**
**WB-3**	6.65	-89	**11.20**	26	24.8	63.6	11.6	**44.7**	4.20	**26.0**	**1040**
**WB-4**	6.62	-86	**10.50**	18	21.0	63.5	15.5	**39.7**	4.13	**98.1**	**2680**
**WB-5**	6.65	-80	**10.20**	12	23.5	66.3	10.2	**37.4**	3.92	**122**	**7660**
**SR-0**	7.03	-169	**0.40**	0.94	6.9	13.6	79.5	**5.5**	3.56	**1.70**	**8.9**
**SR-1**	6.96	-171	**0.60**	0.90	7.9	15.7	76.4	**6.4**	3.56	**11**	**56.6**
**SR-2**	7.03	-165	**0.40**	0.77	7.1	12.7	80.2	**5.0**	3.55	**67.7**	**122**
**SR-3**	7.03	-162	**0.40**	0.70	8.4	13.9	77.7	**6.3**	3.55	**164**	**213**
**SR-4**	7.06	-156	**0.30**	0.51	8.1	12.2	79.7	**5.0**	3.57	**801**	**411**
**SR-5**	7.03	-152	**0.40**	0.46	7.9	14.2	77.9	**5.4**	3.57	**5700**	**941**

Nickel distribution coefficient, Kd = TR-Ni/pore-water Ni

Table modified from Besser et al. 2011.

TM = treatment, ORP = oxidation-reduction potential, TOC = total organic carbon, AVS = acid-volatile sulfide, CEC=cation exchange capacity, OW-Ni = Ni in overlying water, TR-Ni=total-recoverable nickel

### Sediment Tests

Sediment preparation and post-treatment analysis: To minimize exposure of worms to soluble nickel, and to imitate the natural flows of water, sediments were overlaid with excess test water prior to use. Water was exchanged twice daily for one week. For one replicate, the last water exchange was collected and sent to the USGS for analysis to estimate the concentrations of soluble nickel in the pore water. Additionally, the post-treatment test sediment also was sent to the USGS for composition analysis [40].

#### Food preparation

This test used OP 50 *E. coli* bacteria as a food source. 500 mL flasks of OP 50 were inoculated with single colonies from an isolation plate. The bacteria were allowed to grow one day at 37°C and stopped while still in growth phase. Bacterial cultures were removed from the incubator and ampicillin was added. Cultures were allowed to continue for 12 hours to halt bacteria growth and kill all cells. Cells were washed three times to remove media, waste, and antibiotic, and then suspended in test water [OP 50; aqueous test: 1,000 50 formazin absorption units (FAU; according to ISO 7027); sediment and soil test: 12,000 600 FAU]. 2 µL of cholesterol in ethanol were added per ml of *E. coli* suspension. To confirm that bacteria were dead, treated cells were streaked on NGM plates and incubated overnight to insure no growth occurred.

#### Sediment measurement

To dispense an equal volume of sediment for each test, 1 mL of wet sediment was determined for test replicates by weight. To determine the appropriate mass, 20 mL of sediment was placed in a previously tared conical tube and the net sediment weight was divided by 20.

#### Test design

Tests were run based upon modifications of previously established standards [14,15,49]. At least two trials were conducted for every test sediment. In a single trial, six replicate wells were set up. Thus, the recovery result for each individual sediment type represents the cumulative results from a minimum of 120 initially loaded animals. Within individual wells of a 12-well tissue culture plate, 1 mL of sediment and 0.5 mL of OP 50 suspension were added. The sediment and food were mixed using a toothpick, and 10 synchronized, freshly hatched hermaphrodite larvae were added to each well. 12-well plates with fully set-up trials were sealed with parafilm and placed on a rotating platform at 20°C. Tests involving *C. elegans* were left in the incubator for 96 hours and tests involving *P. pacificus* for 120 hours.

#### Adult and progeny recovery

At the end of the test duration, animals were recovered from the sediment for analysis, and 4 mL of a silicate suspension solution was added (1 part Ludox TM-50 colloidal silica: 2 parts H_2_O) to each well. The contents of the well were mixed and transferred to a 15 mL conical tube and centrifuged at 800g for 10 minutes. The sediment pelleted to the base of the tube and adult nematodes and larvae remain suspended in solution. The suspension was drawn off and placed in 100 mm petri dishes. The pellet was suspended twice more in the silicate suspension, then scanned under the stereo dissecting microscope for nematodes. Live adults and recovered larvae were counted and placed into a new tube. Dead animals, mostly larvae, likely P0 animals, were recovered from many types of sediment. Most dead animals recovered showed substantial signs of decomposition; specifically, poor internal morphology of organs due to decomposition and the presence of bacteria in the body cavity/organs outside the lumen of the gut. Because all live animals recovered were adult, most dead animals were larvae, and the corpses recovered were substantially decomposed, dead animals were assumed to have been victims of the treatment and not the recovery process. This is an assumption supported by the high recovery of live animals in un-spiked reference sediments, e.g. WBO. We counted dead larvae as P0 because the size and developmental stage were beyond what is attainable by hatched F1 progeny. Not all P0 corpses were recovered in our washes.

#### Fixation of animals

Collected animals were fixed in a 3% Rose Bengal solution (weight per volume) and baked at 80°C for 10 minutes. Fixed animals assume a rod-like shape allowing easier measurement without altering length and width measurements. Fixed animals were placed on agar pads on microscope slides and a cover glass was added for microscopy and further analysis.

### Water-only Tests

Water-only tests were set up using methods similar to the sediment tests. Food preparation and the setup in the 12-well tissue culture plates were identical with the exception that a 1 mL water sample was substituted for the sediment. At the end of the test, an additional volume of sterile distilled water was added to each well and nematodes were transferred to empty 100 mm petri dishes and counted. Counted nematodes were transferred to a new collection tube and fixed for analysis under the compound microscope as described above.

### Microscopy

Fixed nematodes were analyzed on a Nikon Microphot FX compound microscope with differential interference contrast (DIC) optics. Images were taken using a Nikon DS-Qi1Mc digital camera and NIS-Elements: Basic Research software. Length and width measurements were taken for two independent sets of treatment trials using 200X images with NIS-Elements software. Length was determined by tracing a curve from tip of the head to the tip of the tail along the dorsal-ventral midline of the animal using the gut as a reference. Width measurements were taken at the vulva, a line was drawn on the ventral side on the animal between the anterior periphery of the vulva and posterior periphery of the vulva. Using this line, NIS-Elements produced a perpendicular line bisecting the vulva, from which width measurements were taken. Concurrently, animals were scored for the presence of embryos in the uterus. Life-stages were confirmed based upon vulva and gonad morphology in addition to size and fertility.

### Fecundity Index

All recovered progeny were L1s or early L2/J2s based upon size and gonad morphology. For each sediment and test water treatment, the average number of progeny from each worm within a well, based upon total larval counts at the end of the test, was computed by dividing the total recovered F1 progeny from the well by the total recovered live P0 parental adult animals. For statistical analysis of fecundity, individual ratios for every test well were generated and treated as single data points (i.e. for each sediment or water type n=12).

### Longevity Assays, i.e. Adult Survivorship Curves

To assess the effects of nickel upon adult lifespan, 22 test wells were established (enough to cover a three week period) using the WB nickel-spiked sediment series: WB-0, WB-2, WB-3, and WB-5. *Cel-fog-2* (q71) mutant L4 female larvae were added to each well. *Cel-fog-2* mutants grown in WB-0 showed a normal reproductive lifespan compared to the N2 laboratory strain grown on petri dishes [50–52]; however, hermaphrodites do not make sperm [53], meaning no progeny were produced throughout the duration of our assay. Due to different dates of the trials, slightly different numbers of animals were added to the WB-0 and WB-5 treatments (~55 animals) than the WB-3 and WB-4 treatments (~75 animals). Once a day for 22 days, at the same time each day, adults were recovered from a single well for each sediment treatment. Longevity was quantified via percentage survivorship, calculated as the number of recovered live animals divided by the number of animals initially added into the well. Because the tests were significantly longer and the number of nematodes added greater than for a four-day test, an additional 200 µL of food was added to each remaining test well every five days.

### Statistical Analysis

Statistical analyses were done and graphs generated using IBM program SPSS Statistics version 20. In each figure the graphing package identifies outliers: an open circle indicates that the point is an outlier less than three times the height of the box, whereas a star or asterisk indicates an extreme outlier greater than three times the height of the box. Independent of the graphical representation, a comparison of means among different treatments was performed on the entire data set (including the designated outliers) using one-way ANOVA with Tukey post-hoc comparison tests (*p*≤ 0.05), assuming homogeneity of variance among the populations.

Using least squares regression modeling, best-fit models for P0 recovery as a function of nickel concentration in sediments were first generated using Microsoft Excel 2008 for the Mac version 12.3.4, and subsequently confirmed using both SPSS and Minitab 16 Statistical Software (Table 5). Based upon best-fit equations, a lethal concentration (LC50, 50% lethality of P0 animals over test duration) for substrate bound nickel was estimated for the WB and SR nickel-spiked sediment series. Each formula was intersected by a line through y=5 for *C. elegans* and y=4 for *P. pacificus* in the WB spiked series while a line at y=3 was chosen for *P. pacificus* in the SR spiked series. These y-intercepts represent 50% of the control sediment survival. In order to achieve an exponential decline (i.e., a curve that did not cross the y=0 axis) P0 recovery data points were adjusted by 1 (n+1) and, consequently, the y-intercept also was raised by 1 to estimate the nickel LC50 using the exponential curve. LC50 ranges given in the results section are based on binomial and trinomial curves, as these models consistently gave high R^2^ values.

**Table 5:** Best fit curves for survivorship as a function of sediment nickel concentration.

**Formula**	**Best-fit curve**	**Nickel µg/g**	**R^2^**
**For WB *C. elegans***			
Binomial	Y=5*10^-07^x^2^ -0.0049x + 10.426	1272.60	0.9754
Trinomial	Y=2*10^-10^x^3^ -1*10^-06^x^2^ - 0.0014x + 9.6338	1813.16	0.9965
Log	Y=-2.314ln(x) + 20.796	921.75	0.8291
Exp (n+1)	Y=8.8914e^-3E-04x^	1311.08	0.7106
**For WB *P. pacificus***			
Binomial	Y=4*10^-07^x2 -0.004x + 7.9846	1122.05	0.9326
Trinomial	Y=-1*10^-11^x^3^ + 5*10^-07^x^2^ -0.0042x + 8.0417	1104.27	0.9328
Log	Y=-1.919ln(x) + 16.753	769.43	0.8935
Exp (n+1)	Y=6.8283e^-3E-04x^	1038.79	0.7016
**For SR *P. pacificus***			
Binomial	Y=2*10^-05^x^2^ -0.0224x + 6.9736	221.00	0.965
Trinomial	Y=2*10^-08^x^3^ -2*10^-05^x^2^ - 0.0132x + 6.572	215.43	0.9756
Log	Y=-1.563ln(x) + 11.008	167.92	0.8093
Exp (n+1)	Y=6.5431e^-0.002x^	246.06	0.7625

Best-fit models for *Cel-fog-2* longevity survivorship were generated as a function of days in sediment and used to estimate the time population sizes reached 50% of the initial animals added (Table 6). The average life span in days is based upon the intersection of the formula described for each sediment and a recovery ratio of y=0.5.

**Table 6:** Best fit curves for survivorship as a function of time (days).

**Sediment**	**Trinomial best-fit curve**	**Day**	**R^2^**
**WB-0**	Y= 0.0001x^3^ -0.0056x^2^ + 0.0347x + 0.8407	14.052	0.7985
**WB-2**	Y = -0.0003x^3^ + 0.0108x^2^ -0.1324x + 1.1398	18.172	0.7651
**WB-3**	Y = 0.0001x^3^ -0.0014x^2^ - 0.0633x + 1.0282	7.751	0.8995
**WB-5**	Y = -0.0004x^3^ + 0.0158x^2^ -0.2272x + 1.0489	2.991	0.9702

## Results

### A wide range of sediments were used for our experimental tests to develop nematodes as a model for this study

The suitability of *C. elegans* and *P. pacificus* were tested in growth, fertility, and survivorship assays using a broad range of sediment types collected from sites in the Missouri River, Mississippi River, and Saint Lawrence watersheds (Figure 2). These sediments vary in their physical attributes including total organic carbon (TOC), acid volatile sulfide (AVS), particle size, and cation exchange capacity (CEC) (Table 1). Larger amounts of organic matter increase the ability of sediments to absorb positive ions; thus there is often a direct correlation between TOC and CEC. The sediments and pore waters also were analyzed for constituents, including dissolved organic carbon (DOC), major anions and cations, and trace elements including nickel (Tables 2 and 3).

For sediment assays, 10 freshly hatched synchronized larvae were placed in individual wells, grown for one life cycle, and harvested to analyze nickel’s effects upon growth, fertility, survivorship, and fecundity (see Materials and Methods). Growth was scored based upon length and width measurements, fertility by the presence of eggs in the uterus, survival by live recovery counts from the sediment, and fecundity by the total number of recovered hatched progeny divided by the number of recovered adults.

### *C. elegans* performs best in sediments containing high levels of organic carbon. In contrast, recovery of *P. pacificus* does not correlate with carbon content

Both *C. elegans* and *P. pacificus* grow and can be retrieved from many, but not all of the sediments tested. For the eight sediments tested, all animals added were recovered as either dead carcasses or live fertile adults after four days of growth for *C. elegans*, and five days for *P. pacificus*. All live animals recovered were identified as adults based upon overall size, and gonad and vulva morphology. Thus, none of the sediments tested noticeably impinged upon or delayed nematode development from larvae to adults. Recovered adults universally made fertile gametes as demonstrated by microscopic observation of sperm, oocytes, and fertilized eggs in the uterus (Figure 1). Additionally, measurements of length and width showed no statistical difference among the animals grown in the disparate test sediments (see Table S1), nor did they show morphological differences compared with appropriately staged animals grown in standard laboratory culture.

**Figure 3:**
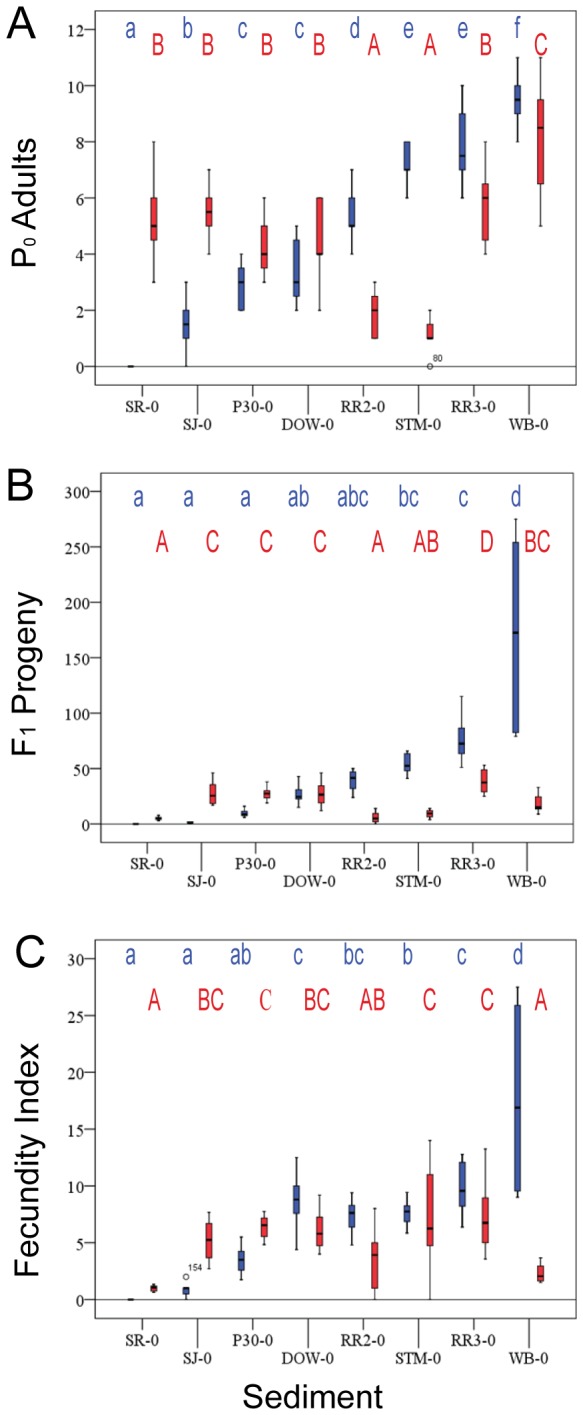
Recovery of P0 adult nematodes and F1 progeny from eight un-spiked test sediments. *C. elegans* – blue. *P. pacificus* – red. Box and whisker plots: box represents the range between the 25^th^ and 75^th^ percentile (interquartile range). The line within the box represents the median. The whiskers indicate minimum and maximum values, except where circles and stars represent outliers (>1.5 times interquartile range from median) or extreme outliers (>3 times interquartile range from median), respectively. Letters above the box and whisker plots represent significant groupings based upon Tukey post-hoc comparison tests (p< 0.05). Lower case blue letters, *C. elegans* groupings; capital red letters, *P. pacificus* groupings. (A) Recovery of added P0 animals. (B) Recovery of L1/J2 progeny. (C) Fecundity Index, total progeny recovered / total live P0 adult nematodes recovered.

*Caenorhabditis elegans*, a free-living soil nematode frequently isolated from the wild in orchards and areas rich with decomposing plant matter, demonstrates a strong preference for sediments rich in organics with associated high cation exchange capacities, mimicking their endogenous natural associations (Table 1 and Figure 3A). In these organically rich sediments, nearly every *C. elegans* hermaphrodite L1 larva added to the sediment was recovered four days later as an adult; in sediment WB-0, which had the highest total organic carbon, ~100% of animals were recovered. Despite their preferences, *C. elegans* can be grown in less organically rich sediments, albeit with lower recovery rates. Only in the SR sediment, which contains the least organic matter, did we fail to recover any adult animals, but several other sediments resulted in less than 50% average recovery. In a statistical analysis of other potential correlations, we found no additional obvious sediment property associated with recovery rates of *C. elegans* adults from the sediments tested (Tables 1-3). The results for individual sediments were consistent across six replicates within each trial and across multiple trials (see Materials and methods).

Also a cosmopolitan free-living nematode, *P. pacificus* is often found associated with specific beetle families and not as frequently in the same areas of rich organic matter as *C. elegans*. Not surprisingly, the recovery profile for *P. pacificus* differs greatly from that of *C. elegans* (Figure 3A). Recovery of this species averaged less than 50% in several sediments. Once again, statistical analyses found no other obvious correlations with any sediment parameter (Tables 1-3). *Pristionchus* consistently performed poorly in terms of adult recovery in some sediments where *C. elegans* performed very well. Likewise in SR-0, a sediment entirely inhospitable to *C. elegans*, *P. pacificus* performed well, with ~60% of animals recovered as live adults.

In addition to adults, larval progeny were recovered. Both *C. elegans* and *P. pacificus* are self-fertile hermaphrodites; upon reaching adulthood they begin to produce progeny immediately. Our four-day sediment tests provide ample time for *C. elegans* to reach adulthood. In many of the sediments, the original animals added to the test wells (the P0 generation) produced F1 progeny. For both our sediment and water tests described below, we calculated the average number of progeny per adult worm recovered over the duration of the tests for each nematode species. We use this number as a reference index of fecundity. Generally speaking, progeny numbers correlate with the number of adults recovered; that is, more adults result in more progeny for *C. elegans* (Figure 3C), and fecundity indices are statistically similar for most sediments. Indices are lower in sediments SR-0 and STJ-0 due to lack of recovery of any or almost any P0 animals. Only in *C. elegans*’ favored WB-0 sediment is the fecundity index significantly higher. *Pristionchus* grows more slowly and, while five days proved ample time to reach adulthood, *P. pacificus* progeny were just beginning to be produced within this time period. Thus, for our *P. pacificus* sediment tests, the F1 progeny numbers are too low to demonstrate a correlation with adult (P0) recovery, although this seems likely (Figure 3B and 3C).

Besides organic carbon and cation exchange capacity for *C. elegans*, adult recovery, fecundity, and life span (presented later in the results) showed no other strong correlations with other parameters in the eight test sediments, including particle size, endogenous metal ions, or other ions. No correlations with high R^2^ were identified using one-, two-, or three-factor modeling. Uncovering further correlations of phenotype with other sediment physical characteristics is likely to require a much broader set of sediment samples to tease out small and complex effects involving multiple characters.

### Exposure to nickel-spiked sediment results in concentration-dependent lethality in *C. elegans* and *P. pacificus* but does not affect growth or fertility

Two contrasting sediments, WB and SR, were chosen to study of the effects of nickel in spiked whole-sediments. Sediment WB has high concentrations of TOC and AVS, and a high cation exchange capacity and, thus, is able to adsorb a relatively high concentration of nickel compared to SR. Both sediments were spiked to obtain five treatments with sequentially greater bound-nickel content, WB-1 through WB-5 and SR-1 through SR-5 (Table 4). Because of its greater nickel-binding capacity, nickel was spiked at much greater concentrations in WB sediments than in SR sediments (Table 4).

**Figure 4:**
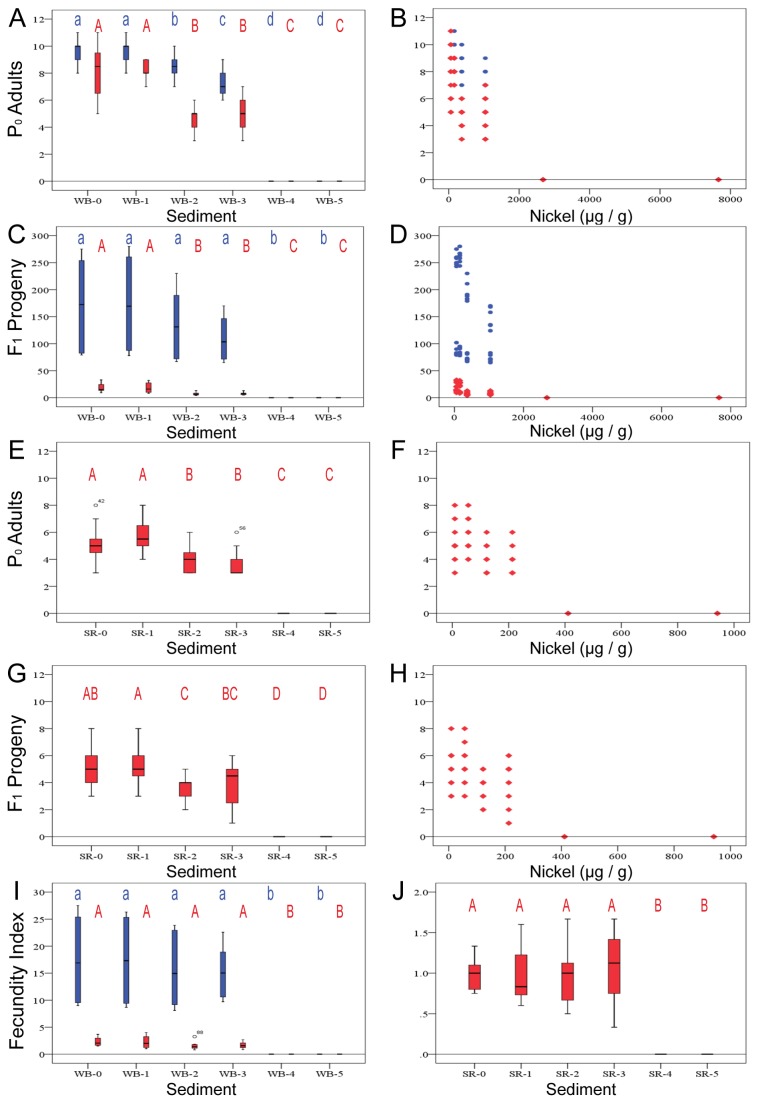
Recovery of P0 adult nematodes and F1 progeny from nickel-spiked sediments (i.e. WB-0 through WB-5 and SR-0 through SR-5). Box-and-whisker plots are formatted and labeled as in Figure 3. Scatterplots: P0 recovery for individual wells plotted against sediment nickel concentration. Blue circles, *C. elegans*; red diamonds, *P. pacificus*. (A-D, I) WB Ni(II)-spiked sediment series. (A) P0 recovery and sediment treatment. (B) P0 recovery and sediment nickel concentration. (C) F1 recovery and sediment treatment. (D) F1 recovery and sediment nickel concentration. (E-H, J) SR Ni(II)-spiked sediment series. (E) P0 recovery and sediment treatment. (F) P0 recovery and sediment nickel concentration. (G) F1 recovery and sediment treatment. (H) F1 recovery and sediment nickel concentration. (I and J) Fecundity ratio and sediment treatment.

Nickel-spiked sediments decreased survivorship of *C. elegans* and *P. pacificus* larvae in a concentration-dependent manner (Figure 4A and B, E and F). For both *C. elegans* and *P. pacificus*, the recovery of adults declined steadily in WB treatments from the un-spiked (WB-0) to WB-3 sediments, and no animals were recovered from spiked treatments WB-4 and -5 (Figure 4A and B). Based upon best-fit analysis, a concentration of nickel resulting in 50% lethality compared to the un-spiked control (LC50) was estimated at between 1,273-1,813 µg nickel per g sediment for *C. elegans*, and 1,104-1,122 µg/g for *P. pacificus*. A similar profile was also observed for *P. pacificus* using the SR sediment series (Figure 4E and F). Because it cannot tolerate the SR environment, no *C. elegans* data were obtained from the SR series. Recovery of *P. pacificus* steadily declined from the un-spiked SR-0 through SR-3 sediments; no *P. pacificus* adults were recovered from sediments SR-4 and SR-5. The estimated LC50 for *P. pacificus* was 215-221 µg/g of SR sediment. Intriguingly, animals appeared to tolerate a much higher amount of total nickel in the high-organic, high-AVS WB sediment than in low-organic, low-AVS SR sediment. This contrast is consistent with relationships between nickel toxicity and sediment constituents reported for other invertebrates [40].

The number of progeny recovered also decreased with increasing sediment nickel concentrations (Figure 4C and D, G and H). This reduction in progeny largely results from a smaller number of the original progeny surviving to adult (reproductive age) than in un-spiked sediments. This is supported by statistically similar fecundity indices from all sediment treatments save those where no P0 adults were recovered (Figure 4 I and J).

### Testing of nematodes in water

**Figure 5:**
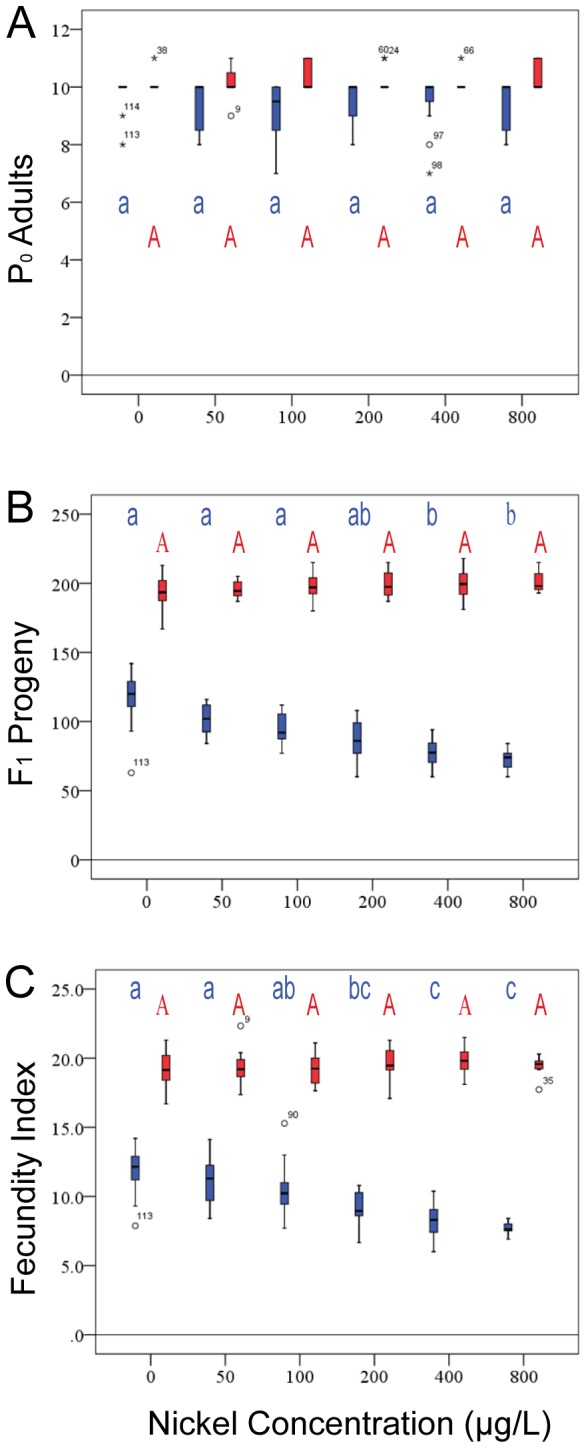
Recovery of P0 adult nematodes and F1 progeny from nickel-spiked water. *C. elegans* – blue. *P. pacificus* – red. (A) P0 recovery. (B) F1 recovery. (C) Fecundity ratio.

In addition to testing sediments we tested moderately hard water analogous to that overlying the eight test sediments. Water controls were set up in an analogous fashion to the sediment tests. Animals added to the control water and subjected to testing universally grew to fertile adults with ~100% recovery after respective growth periods for *C. elegans* and *P. pacificus* (Figure 5A). Therefore, an aqueous environment *per se* had no obvious effect upon the ability of nematodes to survive and reach adulthood.

We also counted larvae, the progeny of our original 10 L1s or J2s. With the possible exception of sediment WB for *C. elegans*, a much larger number of progeny typically can be isolated from water tests than sediment tests (compare Figures 3B and 5B). We do not believe this is due solely to the retention of larvae in the sediment fraction during harvest, as sequential washings with additional silicate suspension yielded quickly diminishing returns. Additionally, we do not observe increased numbers of larval corpses. Rather, based upon time to maturity as indicated by vulva and gonad morphology (see Figure 1), as well as reduced brood sizes produced during the test, we suggest that the original L1 larvae grow more slowly in the sediment than in the fully aqueous environments. Using spot checks in un-spiked water tests, *C. elegans* animals with fully adult vulva morphology and embryos *in utero* could be isolated at 80 hours; no animals with fully mature morphologies were isolated at the same time point from WB0 sediment trials.

### Water-soluble nickel does not affect growth from larva to adult, fertility, or survivorship of the P0 animals, but does affect the production of the F1 generation in both *C. elegans* and *P. pacificus*

Toxicity observed in whole-sediment tests results could be due to soluble nickel in pore water or overlying water. To evaluate the contribution of aqueous nickel to the results from our whole-sediment assays, we determined growth, fertility and survivorship of nematodes in test water spiked with progressively higher NiCl_2_ concentrations (Figure 5A). The maximum nickel concentration used in these assays (800 µg/L) was greater than or equal to nickel concentrations measured in overlying water of all assays with spiked SR sediments, to a maximum of 122 µg/L in SR-5 (Table 4). For all nickel concentrations tested, approximately 100% of *C. elegans* or *P. pacificus* larvae added were recovered as fertile adults, all statistically similar in both length and width (see Table S1). Therefore, differences in P0 recovery observed in our spiked sediment tests are not due to the presence of water-soluble nickel.

We also looked at the number of progeny of both species recovered from our nickel-spiked water tests. Results of the water-only assays indicate that no significant effects on *P. pacificus* would be expected during exposure to aqueous nickel concentrations (e.g., in pore water or overlying water) of 800 µg/L or less, but that significant effects on *C. elegans* reproduction can occur at aqueous nickel concentrations as low as 400 µg/L (progeny, Figure 5B) or 200 µg/L (fecundity, Figure 5C). *Caenorhabditis* has a lower tolerance for soluble nickel than *P. pacificus* in our assay. Our fecundity index indicates the reduction in larval progeny in *C. elegans* reflects a decrease in the number of progeny generated per adult worm (Figure 5C). This could represent a delay in the maturation of the parental germ line, a defect in parental gametes that reduces the rate of progeny production, a developmental delay in the progeny, or embryonic or larval lethality in the progeny. Analyses of the appearance of adult traits during nickel water treatment indicate that adult features such as vulva evagination, gonad arm morphology and gamete production occur at approximately the same time (Figure 1), meaning a delay in parental maturation is not a likely cause. Similarly, we did not recover dead or obviously abnormal larvae, indicating larval lethality does not explain reduced progeny numbers.

### Nickel-spiked sediment decreases the lifespan of adult nematodes in a concentration-dependent fashion

Our four-/five-day larva to adult growth/survival tests for *C. elegans*/*P. pacificus* do not address potential loss of fitness due to reduced adult lifespan or long-term nickel exposure. *Caenorhabditis* hermaphrodites lay ~300 eggs without mating, at an average rate of approximately four eggs an hour over roughly three days [54]. If mated with a male, a hermaphrodite can lay nearly 1000 eggs over a longer time span. Thus a reduced lifespan can have dramatic effects upon the total number of progeny generated and overall fitness.

**Figure 6:**
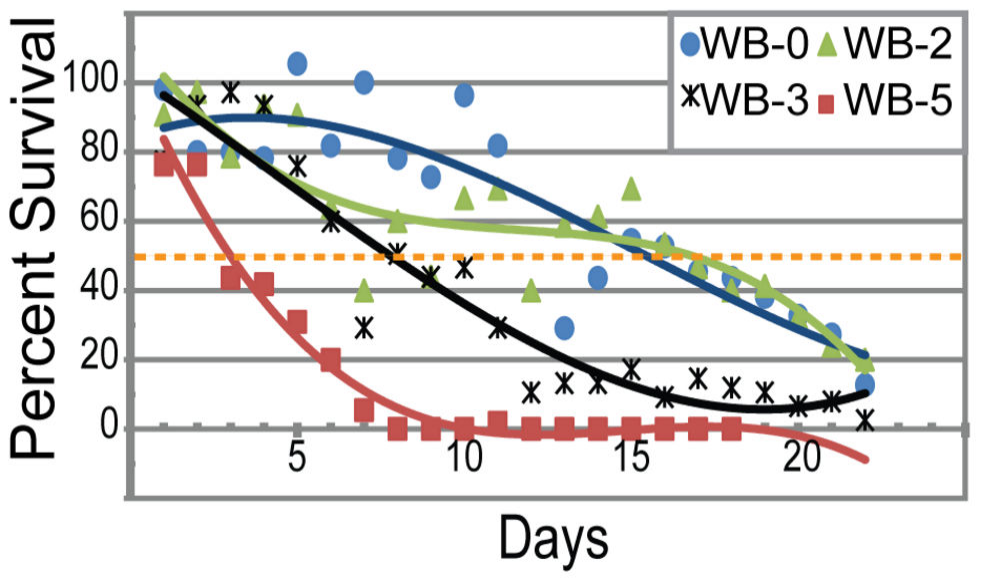
Adult lifespan in nickel-spiked sediment – survivorship curves. Recovery of *Cel-fog-2* females from WB spiked nickel sediments, WB-0 (blue circles), WB-2 (green triangle), WB-3 (black asterisks), and WB-5 (red squares). Survivability decreases as nickel increases. Yellow-orange dotted line represents a 50% recovery. WB-0 and WB-2 show a 50% reduction around day 16. WB-3 shows a 50% reduction around day 7. WB-5 showed a 50% reduction around day 3.

To test whether nickel reduces adult lifespans, we set up 22 wells analogous to those of four-day tests for each of four sediment treatments, WB-0, WB-2, WB-3, and WB-5, and added *C. elegans* fog-*2* hermaphrodite animals to each well (see Materials and methods). The *Cel-fog-2* mutation results in hermaphrodites being transformed into female animals [53]. Compared to N2 animals, they have normal lifespans [50–52] in our tests with un-spiked WB-0 sediment, but do not produce progeny unless mated by a male. We used unmated *C. elegans* L4 *fog-2* females in our longevity assays. As a result, no progeny are produced that could be confused with the original animals added to test wells. Throughout the 22-day duration of the test, we harvested the animals from a single well daily for each treatment. Our results indicate a concentration-dependent relationship for adult life span (Figure 6). Around 50% of the animals were dead after ~16 days in culture using WB-0 and WB-2 treatments. For our assays using WB-3 and WB-5, 50% lethality was reached before day 8 and day 4 respectively. Thus higher nickel levels in whole-sediment assays decreased life span as much as four-fold, dramatically reducing lifetime reproductive potential.

## Discussion

The anthropogenic introduction of metals into the environment is an ongoing concern for both conservation biology and human health. Our results clearly show complex detrimental effects of nickel upon nematodes at multiple stages of their life cycle, and at environmentally relevant concentrations in naturally occurring sediments and water. Additionally, our comparative approach using two ecologically and molecularly diverged nematode species, clearly demonstrates the advantage of applying a broader spectrum of nematode biodiversity to environmental toxicology assays. Our study reveals several important considerations regarding aquatic nickel toxicity. First, even low concentrations of nickel in realistic environments can have some health and developmental impacts. Second, given the tractable nature of nematodes as environmental and health model systems, it would seem wise to expand analyses to additional members of the phylum, adjusting test formats and parameters of the established sediment assays accordingly, to provide test results from a broader range of sediments and soils. Lastly, adjusting standard nematode tests to allow for multiple generations can provide a broader understanding of environmental toxicity effects.

### Nematode diversity and sediment assays

We tested eight sediment samples over a wide range of physicochemical characteristics using two nematodes, *C. elegans* and *P. pacificus*. These species respond to the un-spiked, control sediments differently. *Caenorhabditis* performs best in sediments rich in organic matter, reflecting the known ecological preferences of isolates from the wild for environments with large amounts of rotting plant material. Our observations in sediment assays are consistent with previous findings that the type and state of the organic matter in the sediment elicits specific genetic and physiological responses [55]. Thus, sources of organic carbon also should be considered when using *C. elegans* as a test animal for sediment and soil assays. No clear preference pattern for sediments was obvious from *P. pacificus*, although survivorship of this species was low (<20%) in some sediments. Notably, *P. pacificus* was recoverable in sediments like SR, in which *C. elegans* struggled to survive. This has obvious implications for using nematodes as test organisms. Given the diversity of existing nematode species, the variety of free-living strains already collected, and the relative ease of nematode handling, future experiments should take advantage of the full benefits the phylum offers by matching a test nematode to the sediment of interest.

### Considerations to improve the use of nematode-based sediment and soil assays

Our experiments also suggest that adjustments to the standard test protocols established by international and national monitoring agencies could yield improvements in data collection. Many studies conducted by testing agencies have required >90% recovery from un-manipulated sediments to consider results from the spiked sediments to be relevant. In our tests, *C. elegans* meets these guidelines for only a few sediments and *P. pacificus* perhaps not at all. We found, however, that despite lower recovery in many of the sediments, rates were quite reproducible and yielded highly reliable statistical comparisons in the context of our relatively large sample sizes. This was a benefit of our use of larger numbers of nematodes per well and greater numbers of trials per test condition than required by many mandated protocols. Our data largely agree with the reproducibility of sediment and soil sample trials carried out in parallel in separate labs [16]. These findings suggest that the use of a combined ‘control-plus-reference’ approach, with sample sizes higher than currently required by international monitoring guidelines, should be adopted for use with nematodes. In this design, sediments known to be suitable for the nematode of choice would be selected to serve as a control to document baseline nematode health under test conditions. Several uncontaminated sediments representative of the study area would then be selected to serve as reference sediments, against which the toxicity of contaminated sediments could be evaluated.

### The effects of nickel upon nematode developmental survival and growth

Our data demonstrate a concentration-dependent effect of nickel in sediments on nematode survival from larva to adult. Survivorship steadily decreased for both *C. elegans* and *P. pacificus* as nickel concentrations increased in WB sediments. The same held true for *P. pacificus* in sediment that is inhospitable to *C. elegans*. Although the effects of nickel we observed are comparable to those seen in other organisms, we found nematodes to be more sensitive to lower exposure levels, at concentrations similar to anthropenically derived nickel [2], than has been reported for many other orders of animals [40]. This further underscores the value of nematodes as a model animal for toxicity studies.

In whole-sediment tests, toxic effects on nematodes could result from exposure to both substrate-bound nickel and soluble nickel in pore water. In our studies, test water treatments were spiked with NiCl_2_ to approximate the concentrations in the overlying water of lethal nickel-spiked sediments. The absence of significant lethality in our water-only test suggests that the lethality we observed in whole-sediment tests is primarily due to sediment-bound nickel. Further, the LC50s for *C. elegans* and *P. pacificus* in the WB sediment were very similar, although *C. elegans* showed a slightly higher tolerance to substrate-bound nickel in our survivorship assays. Our data suggest that substrate-bound nickel is more lethal to nematodes, perhaps mimicking insoluble nickel compounds and their modes of action.

Sediment composition appears to have a substantial impact upon nickel bioavailability [33]. Organic carbon is a large component of sediment WB, whereas sediment SR has very little organic carbon. Organic carbon binds divalent metal ions very well; thus, WB has a high cation exchange capacity. Although our study shows no correlation with acid volatile sulfides, binding of particulate nickel in the form of nickel sulfide increases CEC and could affect bioavailability. Based upon lethality results for *P. pacificus*, WB sediment required addition of a much higher nickel concentration to cause total lethality compared to SR. Because sediment WB binds nickel better, it is more likely to retain it for longer periods. Although this finding is confounded by generally high mortalities seen even in control SR sediments (Figure 3A), the differences in *P. pacificus* LC50s estimated for SR and WB sediments (Table 5) are consistent with the large differences in nickel toxicity thresholds between spiked SR and WB sediments for other invertebrates [40].

Despite previous results suggesting that nematodes grow larger upon exposure to high concentrations of aqueous nickel [35], we did not observe an effect of nickel upon adult body size. The highest concentration of nickel evaluated in our water-only tests was 3.4 µM (800µg/L). As a previous study showed only modest effects on adult body size at low concentrations of nickel, but more substantial effects at higher concentrations (75 or 200 µM), it is possible that our studies did not reach the threshold of nickel required to impact growth. Given our survivorship data indicating that insoluble nickel can be more toxic than soluble, it would be interesting to see if insoluble nickel (which was not tested in the previous study) would impact nematode body size even more substantially.

### The effects of nickel upon fecundity

Our results demonstrate important negative effects on nematode fecundity in the presence of increasing doses of nickel. While there could be a slight decrease in fecundity per animal recovered as substrate-bound nickel increases, most of the reduction observed can be explained by reduced numbers of recovered adults (Figure 4I, 4J). In water-only tests, however, the cause of decreased progeny is not related to the number of recovered adults, as essentially all adults are recovered from each test. In this series of tests, the average number of progeny per adult recovered over the lifetime of the test decreased as NiCl_2_ concentrations increased. This reduction in offspring could have several causes. First, progeny are not produced until a specific developmental stage. Perhaps it takes nematodes in spiked waters and sediments longer to reach maturity; thus, they produce fewer progeny during the test period. An alternative possibility is that animals reach reproductive age in the same amount of time, but fewer viable progeny are produced. In the examination of staged animals throughout a four-day water test, animals grown in control water and animals grown in 800 µg/L NiCl_2_ exhibited no obvious morphological differences based upon size, gonad morphology, or vulva morphology. Thus, we tend to favor the latter hypothesis, that nickel is having an adverse effect upon gamete production or egg-laying itself.

Why is there a difference in nematode fecundity between water and sediment tests? This is surprising; given the strength of the effects of sediment-bound nickel compared to soluble nickel upon lethality it might be expected that sediment bound nickel would also affect fecundity more strongly. We observed that nematodes grow more slowly in sediment than in solution; thus, they reach maturity later in sediment and have less time to lay eggs. Sediment tests yielded very few larvae, which did not permit rigorous statistical analyses. We hypothesize a test of longer duration, allowing the generation of a larger data set of progeny, would permit the detection of a decline in fecundity due to nickel concentrations in sediments. Though, we cannot rule out physiological alternatives as well. Gamete production and egg-laying involve highly regulated processes sensitive to environmental and physiological cues [18,54]; animals in sediments could simply have a decreased rate of egg-laying compared to animals in waters.

Based upon the lack of a response in fecundity from increased amounts of aqueous nickel during the water test, it appears that *P. pacificus* is less susceptible to some nickel’s effects than is *C. elegans*. This contrasts with sediment survivorship results where *C. elegans* appears to be slightly less susceptible than *P. pacificus*. Thus the severity of nickel’s effects upon different parameters within a species, such as survival and fecundity, are not necessarily linked. These potential differences between species could give insights into the physiological and molecular underpinnings of nickel’s actions.

Given nickel’s known genotoxic and epigenetic effects [3,10], we are particularly interested in its potential to cause defects in gametogenesis and the production of viable offspring. In vertebrates, nickel is known to have an impact on sperm quality and motility, particularly in mammals [56–58]. It seems likely that some issues concerning gamete quality, if not motility [59,60], are very likely to be functionally related in nematodes. Previously, a heritable delay in the generation of the first progeny and decreased brood sizes were noted in nickel sulfate (NiSO_4_) treated parents, without any obvious morphological defects in parental reproductive organs [35]. Further, nickel increases the number of apoptotic cells in the developing germ line [36], supporting its role in causing genomic and/or physiological damage to nematode gametes. Thus, the impact of nickel upon nematodes is not simply straightforward lethality during larval to adult development. Our water-only tests indicate an effect upon either the generational timing of the brood or the actual brood size of the next generation, as shown by previous work [35]. Based upon these earlier studies, many of these effects of nickel could be heritable and, therefore, have multigenerational consequences [35]. Likewise, our measurement of adult lifespan indicates that nickel dramatically decreases both the potential for animals to produce self-fertilized offspring, and opportunities for mating events with males over the lost lifespan. Studying the potential effects of aqueous and substrate-bound nickel upon population size and health clearly requires the development of more sophisticated, longer duration tests, but could yield important findings that apply directly to human health.

## Supporting Information

Table S1 **Length and width measurements from sediment and water.**
(DOCX)
