# Supplementary material for: Assaying Environmental Nickel Toxicity Using Model Nematodes
Source: PLoS One. 2013 Oct 7;8(10):e77079. doi: 10.1371/journal.pone.0077079 (PMC3792034; doi:10.1371/journal.pone.0077079)
Supplement: Table S1 — Length and width measurements from sediment and water. (DOCX) [file pone.0077079.s001.docx]

| **Supplemental Table 1. Length and width measurements from sediment and water.** | | | | | | | |
| --- | --- | --- | --- | --- | --- | --- | --- |
|  | | **Sediment** | **Average Length ± STD (µm)** | **Length Range** | **Average Width ± STD (µm)** | **Width Range** | **N** |
|  | | | | | | | |
| ***C. elegans* Sediment Test** | **Eight sediments** | SR-0 | - | - | - | - | 0 |
|  |  | SJ-0 | 1247.90 ± 153.14 | 1002.12 - 1490.31 | 87.01 ± 11.56 | 62.19 - 107.40 | 19 |
|  |  | P30-0 | 1325.80 ± 128.75 | 1038.66 - 1467.34 | 83.53 ± 12.03 | 53.22 - 103.21 | 35 |
|  |  | DOW-0 | 1283.84 ± 90.02 | 1103.78 - 1498.30 | 85.02 ± 12.58 | 50.32 - 105.47 | 40 |
|  |  | RR2-0 | 1292.89 ± 114.96 | 1113.68 - 1502.61 | 83.19 ± 13.22 | 55.38 - 102.01 | 64 |
|  |  | STM-0 | 1351.38 ± 143.21 | 1101.73 - 1599.19 | 82.26 ± 15.70 | 52.72 - 105.11 | 87 |
|  |  | RR3-0 | 1298.30 ± 130.47 | 1081.04 - 1529.93 | 86.26 ± 11.47 | 59.32 - 105.47 | 94 |
|  |  | WB-0 | 1370.01 ± 140.27 | 1020.83 - 1634.71 | 88.01 ± 18.01 | 45.34 - 135.39 | 114 |
|  |  | | | | | | |
|  | **WB nickel-spiked** | WB-1 | 1310.85 ± 129.65 | 1103.63 - 1530.52 | 70.09 ± 8.40 | 52.24 - 88.46 | 117 |
|  |  | WB-2 | 1364.34 ± 115.67 | 1087.54 - 1575.01 | 73.09 ± 16.41 | 52.11 - 125.20 | 102 |
|  |  | WB-3 | 1350.12 ± 120.42 | 1021.67 - 1504.81 | 78.41 ± 17.80 | 47.68 - 99.41 | 86 |
|  |  | WB-4 | - | - | - | - | 0 |
|  |  | WB-5 | - | - | - | - | 0 |
|  | | | | | | | |
| ***P. pacificus* Sediment Test** | **Eight sediments** | SR-0 | 985.48 ± 124.61 | 725.42 - 1180.58 | 72.15 ± 9.98 | 63.22 - 92.71 | 67 |
|  |  | SJ-0 | 1020.34 ± 115.21 | 734.23 - 1202.66 | 78.71 ± 11.82 | 64.77 - 86.32 | 65 |
|  |  | P30-0 | 991.25 ± 110.41 | 801.12 - 1261.72 | 82.11 ± 9.74 | 60.55 - 102.42 | 52 |
|  |  | DOW-0 | 952.17 ± 152.87 | 762.67 - 1182.07 | 77.51 ± 10.73 | 57.32 - 88.37 | 54 |
|  |  | RR2-0 | 1015.82 ± 130.40 | 742.42 - 1163.82 | 76.44 ± 12.42 | 61.82 - 87.41 | 22 |
|  |  | STM-0 | 1012.63 ± 122.71 | 782.18 - 1242.31 | 81.10 ± 9.82 | 67.72 - 95.29 | 14 |
|  |  | RR3-0 | 989.33 ± 123.78 | 771.42 - 1212.47 | 76.23 ± 10.27 | 61.44 - 89.08 | 68 |
|  |  | WB-0 | 934.84 ± 117.02 | 761.11 - 1210.81 | 75.87 ± 9.18 | 61.18 - 95.57 | 98 |
|  |  | | | | | | |
|  | **WB nickel-spiked** | WB-1 | 1007.02 ± 138.37 | 752.30 - 1205.41 | 78.15 ± 11.23 | 61.02 - 95.64 | 98 |
|  |  | WB-2 | 1024.43 ± 150.35 | 761.99 - 1210.64 | 77.27 ± 9.64 | 61.33 - 94.53 | 57 |
|  |  | WB-3 | 1006 ± 125.22 | 780.21 - 1195.41 | 75.26 ± 10.07 | 55.28 - 96.08 | 59 |
|  |  | WB-4 | - | - | - | - | 0 |
|  |  | WB-5 | - | - | - | - | 0 |
|  |  | | | | | | |
|  | **SR nickel-spiked** | SR-1 | 967.02 ± 142.71 | 752.30 - 1175.41 | 70.66 ± 15.23 | 57.72 - 90.41 | 66 |
|  |  | SR-2 | 993.83 ± 120.35 | 781.99 - 1220.02 | 73.27 ± 10.77 | 62.09 - 98.12 | 51 |
|  |  | SR-3 | 972.88 ± 141.22 | 730.21 - 1871.92 | 83.77 ± 12.41 | 72.88 - 100.10 | 43 |
|  |  | SR-4 | - | - | - | - | 0 |
|  |  | SR-5 | - | - | - | - | 0 |
|  | | | | | | | |
|  | | **Ni Water (µg / L)** | **Average Length ± STD (µm)** | **Length Range** | **Average Width ± STD (µm)** | **Width Range** | **N** |
| **Hard Water Test** | ***C. elegans*** | 0 | 1313.92 ± 121.36 | 1103.78 - 1605.38 | 68.62 ± 10.55 | 50.05 - 93.46 | 117 |
|  |  | 50 | 1302.59 ± 127.76 | 1081.04 - 1577.04 | 68.32 ± 10.59 | 49.01 - 92.41 | 112 |
|  |  | 100 | 1320.72 ± 125.58 | 1102.98 - 1571.78 | 67.61 ± 9.79 | 42.35 - 89.90 | 110 |
|  |  | 200 | 1313.02 ± 124.21 | 1098.67 - 1534.72 | 67.66 ± 7.43 | 50.87 - 87.05 | 114 |
|  |  | 400 | 1305.47 ± 130.52 | 1103.76 - 1501.44 | 66.23 ± 8.41 | 55.72 - 84.12 | 114 |
|  |  | 800 | 1319.51 ± 121.63 | 1104.62 - 1602.19 | 68.52 ± 10.93 | 49.03 - 93.41 | 113 |
|  |  | | | | | | |
|  | ***P. pacificus*** | 0 | 1021.41 ± 110.41 | 776.37 - 1204.53 | 69.01 ± 9.77 | 55.72 - 85.31 | 121 |
|  |  | 50 | 1000.89 ± 146.21 | 752.44 - 1200.12 | 66.22 ± 10.40 | 52.16 - 83.08 | 122 |
|  |  | 100 | 1033.08 ± 150.32 | 770.62 - 1192.10 | 68.51 ± 10.02 | 56.28 - 84.81 | 124 |
|  |  | 200 | 1007.36 ± 123.33 | 767.90 - 1183.72 | 65.02 ± 9.32 | 54.23 - 80.17 | 122 |
|  |  | 400 | 1018.42 ± 131.66 | 780.21 - 1210.83 | 61.83 ± 12.29 | 55.61 - 82.33 | 121 |
|  |  | 800 | 1014.87 ± 152.71 | 765.42 - 1215.22 | 65.08 ± 9.83 | 55.58 - 86.74 | 124 |
| Shown are the length and width data for recovered adults from two sets of test, i.e. approximately 120 hermaphrodite animals originally added into 12 totals wells. | | | | | | | |
